# The association between motor coordination impairment and altered functional connectivity among autistic children

**DOI:** 10.3389/fped.2026.1711271

**Published:** 2026-02-10

**Authors:** Muqing Cao, Chengkai Jin, Jin Jing

**Affiliations:** 1 Department of Sport Medicine, Faculty of Sport and Health, Guangzhou Sport University, Guangzhou, China; 2 Department of Maternal and Child Health, School of Public Health, Sun Yat-sen University, Guangzhou, China

**Keywords:** autism spectrum disorder, functional connectivity, independent component analysis, motor coordination, resting-state functional MRI

## Abstract

**Background:**

Motor coordination impairment among children with autism spectrum disorder (ASD) has recently gained increasing attention. However, the relationship between functional connectivity (FC) alterations and motor coordination impairment among ASD remains inconclusive.

**Methods:**

We evaluated motor coordination function using the Developmental Coordination Disorder Questionnaire (DCDQ) and acquired resting-state functional magnetic resonance imaging (rs-fMRI) scans from 23 autistic individuals and 25 typically developing (TD) controls (6–10 years old). Within- and between-network FC was estimated using group independent component analysis (ICA) and group comparison was addressed using two-sample t-tests. Relationships between abnormal FC and motor coordination among ASD were investigated with multiple linear regression, with age, gender, and intelligence quotient (IQ) considered as covariates.

**Results:**

In the ASD group, 1) FC within the right cerebellar crus II was negatively correlated to the score of general coordination (*β* = −.566, *p* = 0.035) and control during movement (*β* = −0.529, *p* = 0.026); 2) FC between the cerebellar network and frontal-temporal-parietal network was negatively correlated to the score of general coordination (*β* = −2.610, *p* = 0.006); 3) Increased FC between the cerebellar network and insular network was associated with a higher score of fine motor/handwriting (*β* = −0.529, *p* = 0.026).

**Conclusions:**

We confirmed the role of the insular network in interoception and motor processing among ASD, which was related to impaired information integrating, relaying, and visual feedback during movement. A significant relationship between the cerebellar network and frontal-temporal-parietal network in motor coordination indicated that a deficit in the planning of movements may contribute to atypical motor skills. The study gained an understanding of neuroimaging traits among ASD children and may provide evidence for the design of the motor-related intervention.

## 1 Introduction

Regardless of difficulties with social interaction and communication and restricted and repetitive behaviors, children with autism spectrum disorder (ASD) often suffer from motor coordination impairment, with a risk of up to 85% (1). Limited motor function of these children results in less interaction with the surrounding environment and people (2), which contributes to rigid behavior and lacking social skills (3), thus affecting their intervention and education (4) and compromising their quality of life (5). For this reason, researchers have suggested that motor function impairment should be listed as the third symptom cluster of autism (6). However, the underlying neuroimage traits of motor coordination impairment in ASD remain inconclusive.

Neuroimage studies have provided much evidence of abnormal neural circuits of ASD, especially those associated with the core symptoms, which supported behavior intervention, thus it is important to investigate the neuroimage traits of motor coordination deficit among ASD for the same reason. Although most of the research reported that motor coordination impairment is related to atypical activation, functional connectivity, and volume change in various brain regions of ASD, the limitations of these studies compromised the generalization of the results (7–22). In Supplementary Table S1, we listed previous research investigating motor dysfunction and neuroimaging changes of ASD. It shows most of the studies had a sample with a large age range which covered preschool children to the middle age population (7, 10–17, 19–21). Given that ASD is an early-onset disorder, the participation of adults might reflect causal, compensatory, or age-related differences in brain FC instead of disorder-related differences, which made the explanation of results complicated (23). ASD is characterized by abnormalities of distributed functional networks, rather than focal impairment (11). However, studies examining the altered within- or between-network FC in ASD are lacking, and most of them were task-based (8, 12, 21), which only reveals brain activity and connectivity elicited by a particular task in an experimental environment (24). Motor coordination relies on the integrated function of distributed brain networks. Task-based fMRI studies in ASD have consistently implicated dysfunction in key circuits, including the sensorimotor network (responsible for basic motor execution and sensory feedback) (21), the cerebellar network (critical for motor timing and coordination) (11), and the fronto-parietal network (involved in motor planning and cognitive control) (12, 17). For example, during visuomotor tasks, individuals with ASD often show fine motor deficits along with atypical activation of posterior parietal, premotor, and striatal circuits involved in translating sensory feedback information into precision motor behaviors (21). And in precise control task, greater ALFF in cerebellar vermis VI was associated with less visuomotor variability of ASD population (11).

While these task-based studies pinpoint activation abnormalities during specific actions, resting-state functional magnetic resonance imaging (rs-fMRI) allows the characterization of intrinsic properties of regional- and network-level functional activation and connectivity (25). Given that some autistic individuals can hardly cooperate with task-based paradigms due to their low intelligence, most of the task-based functional magnetic resonance imaging (tb-fMRI) studies recruited ASD with normal intelligence, which made the results less representative (26). While rs-fMRI is less demanding and more suitable for them and can be performed during sleep and anesthesia as well (27, 28). Altered intrinsic connectivity within or between these motor-related networks may represent a fundamental neural trait that underpins the motor deficits observed in ASD, even in the absence of an explicit task.

Further, independent component analysis (ICA) is widely used in rs-fMRI data analysis. It is a data-driven technique that decomposes mixed rs-fMRI data into independent components (ICs), within which resting-state networks can be found according to their spatiotemporal characteristics (29, 30). It avoids bias caused by prior seed region selection and increases sensitivity to detect inter-individual differences (31). Therefore, to better understand the neuroimage traits and their relationship with motor coordination function in ASD, we used behavior evaluation, rs-fMRI scanning, and whole-brain group ICA in the current research.

Comparison between groups and brain-behavior analysis were both applied to explore the 1) behavior and neural traits of motor coordination impairment between school-aged ASD and typically developing (TD) children, 2) association between altered inter-/intra-FC of different neural networks and motor coordination impairment in children with ASD. Based on the aforementioned literature, we hypothesized that: 1) Compared to TD children, children with ASD would exhibit reduced within-network FC in the sensorimotor and cerebellar networks. 2) Within the ASD group, the severity of motor impairment, as measured by the DCDQ total score, would be negatively correlated with the strength of FC between the cerebellar network and prefrontal/parietal cortical regions. ASD and TD children aged 6–10 years were recruited and completed an rs-fMRI image acquisition. The motor coordination function, intelligence quotient (IQ), and sociodemographic characteristics were evaluated by validated parent-reported questionnaires or observational scales. This study adds to the literature by studying the neural image traits and the association of motor and within-/between- network FC among autistic children. To the best of our knowledge, this is the first study that combined clinical motor measurement of autism and network-level functional connectivity derived by independent component analysis (ICA).

## 2 Methods

### 2.1 Study population and procedure

**Figure 1:**
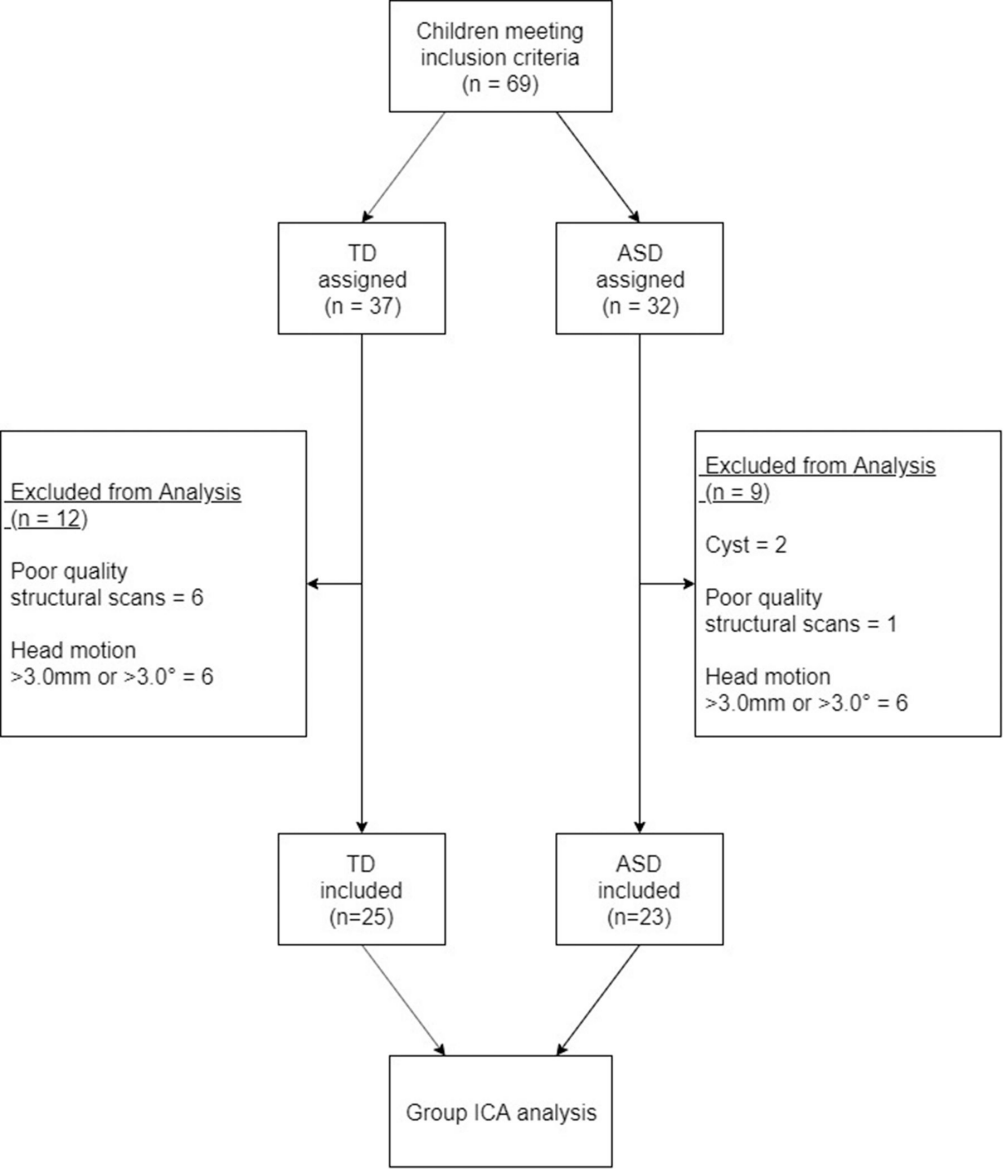
Participant enrolment and exclusion chart.

This study is a part of the ongoing study “the Guangzhou Longitudinal Study of Autistic Children”, which aimed to explore the developmental trajectories of school-aged children with ASD. Autistic children were included if they had a historical diagnosis of ASD which was further confirmed by the Childhood Autism Rating Scale assessment (CARS) and evaluation by two professional child psychiatrists (Jin Jing and Xiu-Hong Li) using the Diagnostic and Statistical Manual of Mental Disorders Fifth Edition (DSM-5). A group of TD children (age-matched) was also recruited at the same time. Participants with a physical handicap or with comorbidity of other neurodevelopmental disorders (e.g., attention-deficit/hyperactivity disorder) were excluded. Participants taking medications known to significantly alter neural activity (e.g., psychostimulants for ADHD, atypical antipsychotics) were excluded from participation to minimize potential confounding effects on functional connectivity measures. All children in this study finished the assessment and MRI data acquisition between Jan 2021 and Feb 2022 from the Center for Child and Adolescent Psychology and Behavioral Development of Sun Yat-sen University, Guangzhou, China. In total, there were 23 autistic children and 25 TD children who had completed behavior and neuroimage data included in the analysis (Figure 1). The study was approved by the Ethics Committee of Sun Yat-sen University, and written informed consent was signed by the caregivers of the children.

### 2.2 Motor coordination function assessment

Motor coordination function was evaluated by the Developmental Coordination Disorder Questionnaire (DCDQ) (32), a 17-item parent-reported questionnaire filled in by the primary caregiver. This five-point Likert scale focuses on various motor skills, which are categorized into three subscales: fine motor/handwriting, general coordination, and control during movement). DCDQ is considered a reliable screening tool for detecting children who are at risk for DCD (33), which was validated with the Movement Assessment Battery for Children (34). It provides a total score ranging from 17 to 85, and three subscale scores with higher scores indicating better motor coordination.

### 2.3 Image acquisition and data preprocessing

Before scanning, the participants were invited to watch a cartoon video introducing the whole procedure of scanning with their caregivers, and researchers further explained the procedure to the children adequately to reduce their nervousness. This increased participants' cooperation and bettered the quality of image acquisition. Children with ASD were administered 0.5% chloral hydrate 0.5 mL/kg (maximum dose 10 mL) orally to induce and maintain sleep because they could not maintain still. The sedation was performed by a nurse trained and certified to administer sedation, following guidelines and protocols. The TD children were asked to stay still with their eyes closed and not sedated considering ethics. A caregiver for each participant was present throughout the entire scan inside the scanner room.

All brain imaging was performed on a Siemens MAGNETOM Skyra 3.0T MRI machine. A T1 anatomical image was collected for co-registration and anatomic localization (TE: 2.19 ms, TR: 1,800 ms, slice number: 176, slice thickness: 1 mm, FOV: 256 mm, matrix: 256 × 256, scan time: 7 min). Echo-planar imaging was conducted to acquire resting-state functional MR data (TE: 30 ms, TR: 2000ms, slice number: 32, slice thickness: 3.5 mm, FOV: 224 mm, matrix: 224 × 224, scan time: 8 min).

Data were preprocessed using the Graph Theoretical Network Analysis software package GRETNA (35). The steps included: (1) Data were converted from DICOM to Nifti format; (2) Removing the first 10 images; (3) Slice timing to correct for the differences in the acquisition time between different slices of a volume; (4) Realign the fMRI images for each subject to correct for head movement, and those with excessive head movement during the scan (>3.0 mm translation and/or 3.0° rotation) were excluded from further analysis; (5) Normalization to warp images into standard Montreal Neurological Institute (MNI) space; (6) Spatially smoothing the images using a Gaussian Kernel (FWHM = 6 mm).

### 2.4 Group ICA analysis

**Figure 2:**
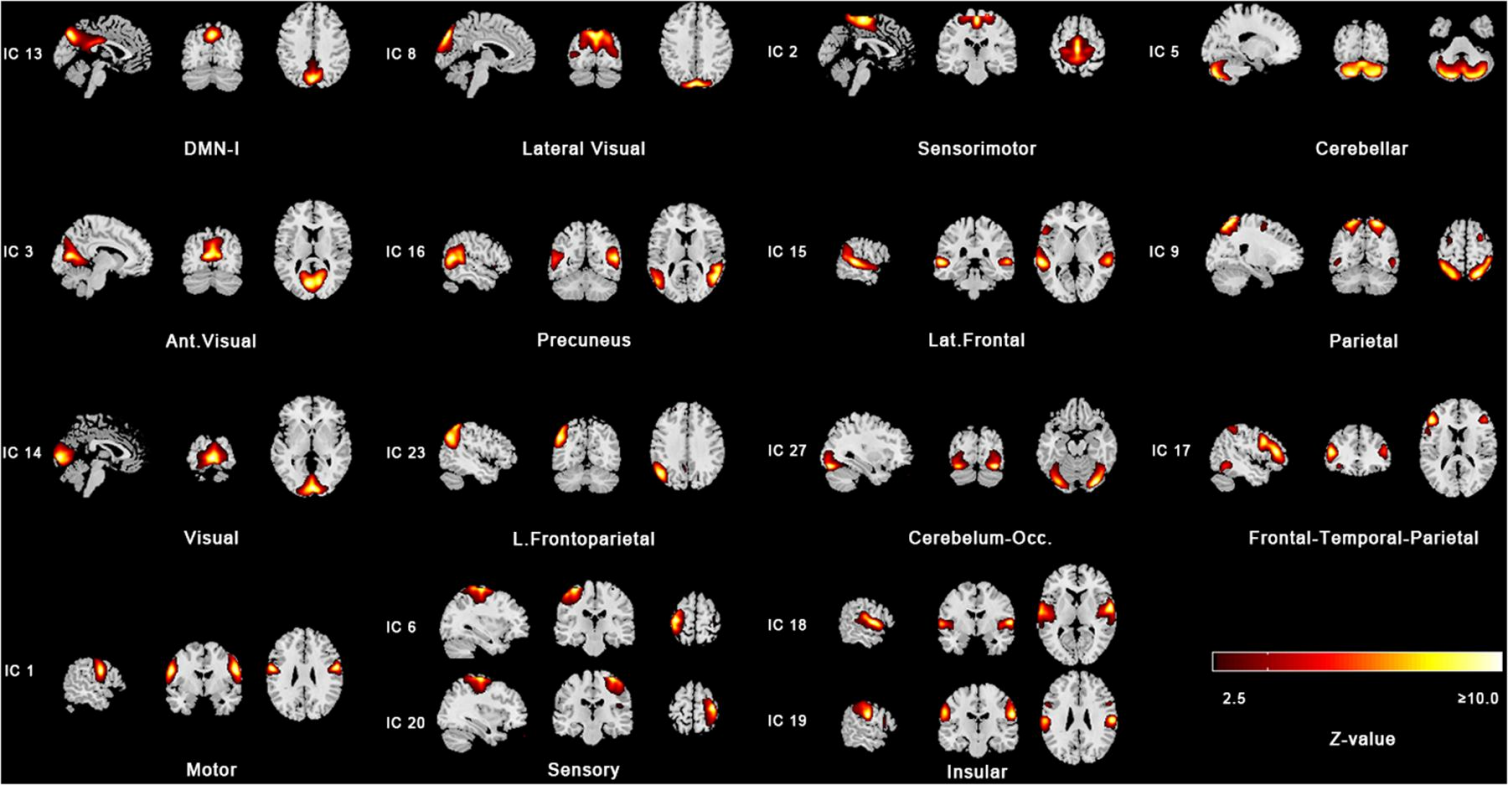
The 15 resting-state networks obtained using independent component analysis. Z-stat maps are thresholded at z = 2.5 and shown in radiological convention.

The processed images were analyzed with the Group ICA of fMRI Toolbox (GIFT, https://www.icatb.sourceforge.net). We used spatial group independent component analysis to obtain functionally connected brain networks. The fMRI data from all the subjects was concatenated to reduce the dimensions using principal component analysis, and independent components (ICs) were estimated and extracted using the infomax algorithm. ICA was applied 20 times to ensure the stability of the decomposition using ICASSO (36). The mean group components were then back-constructed into single subject space, and finally, the fMRI images for all the subjects were decomposed into 35 spatially ICs. Next, the group-level ICs were compared with a pediatric template of resting-state networks (RSNs) to identify different brain networks (37). Those yielded significant spatial correlation (Pearson's *r* > 0.3) with one of the RSNs carried over to the group comparison (38). Overall, 15 networks consisting of 17 ICs were carried over for further analysis: default mode network I (DMN-I), motor, sensory, sensorimotor, cerebellar, precuneus, lateral frontal (Lat. Frontal), parietal, left frontoparietal (L. Frontoparietal), cerebellar-occipital, insular, frontal-temporal-parietal, visual, anterior visual (Ant. Visual), and lateral visual (Figure 2). After these steps, for each subject, we obtained a spatial z-map and its corresponding time course for each IC. For each identified independent component, the value of each voxel in its spatial Z-map represents the strength of functional connectivity between that voxel and the temporal signature of the entire network. Consequently, group differences in within-network functional connectivity are assessed by comparing these spatial Z-maps across groups.

### 2.5 Within-network FC

For each participant, the spatial maps of 17 ICs indicating the within-network FC were extracted. For each component, the participants' z-maps were analyzed using two-sample t-tests with age, gender, and IQ as covariates to examine alterations in within-network FC in ASD (cluster-level *p* < 0.05, corrected by false discovery rate). To ensure that only highly connected regions were analyzed, for each component we used an explicit mask created with the results of the one-sample t-tests of all participants (cluster-level *p* < 0.05, corrected by false discovery rate). The mean FC values of clusters with significant group differences were extracted for further brain-behavior analysis.

### 2.6 Between-network FC

Before analyzing the between-network FC, the preprocessed data were further detrended, regressed of 6 head motion parameters, and band-pass filtered with a Butterworth filter (0.008–0.15 Hz) (39). Then Pearson correlations were computed between the mean time series of the 15 networks for each participant using the Functional Network Connectivity (FNC) Toolbox (implemented in GIFT software). This resulted in 105 between-network correlations for every participant, and they were normalized using Fisher's r-to-z transformation. The Mancovan Toolbox v1.0 (implemented in GIFT software) was used to examine alterations in between-network connectivity in ASD, with age, gender, and IQ as covariates (*p* < 0.05, corrected by false discovery rate). The FC values with significant group differences were extracted for further brain-behavior analysis.

### 2.7 Brain-behavior analysis

We examined the relationship between the abnormal within-network/between network FC and DCDQ scores among all ASD participants using multiple regression analyses, with age, gender, and IQ considered as covariates. The DCDQ scores were standardized (z-score).

### 2.8 Basic characteristics and covariates

Sociodemographic characteristics such as age, family income, education level of parents, and the clinical history of children with ASD such as birth date, gender, date of diagnosis, type of diagnosis, and intervention history were obtained by parent-reported questionnaire. Core symptoms of autism were evaluated by the Repetitive Behavior Scale-Revised (RBS-R) (40) and the Social Responsiveness Scale (SRS) (41). The covariate included in this study was cognition level, which was evaluated by the Wechsler Intelligence Scale for Children-Fourth Edition Chinese version (WISC-IV).

### 2.9 Statistical analysis

Statistical analyses were performed with R Statistical Software (v4.1.2). Differences in demographic and clinical characteristics between the ASD and TD groups were explored with two-sample t-tests (continuous data) or the chi-square test (categorical data), as appropriate.

## 3 Results

### 3.1 Demographic and clinical characteristics

The demographic and clinical characteristics of the subjects are shown in Table 1. Compared with the TD group, the ASD group contained more male participants (82.6% vs. 44.0%, *p* = 0.006) and had a lower full-scale IQ (97.87 ± 17.57 vs. 112.96 ± 10.94, *p* = 0.002), while the age, handedness and mean FD were well balanced. The ASD group also got higher scores in SRS and lower scores in DCDQ (all *p* < 0.001).

**Table 1:** Participant characteristics.

Participant characteristics	ASD, *n* = 23	TD, *n* = 25	*p*	Group Difference
Demographic				
Age, years	7.52 ± 1.27	8.24 ± 1.33	0.063	
Full-scale IQ	97.87 ± 17.57	112.96 ± 10.94	<0.001	ASD < TD
Head motion during rs-fMRI scan, mean_FD_	0.07 ± 0.05	0.10 ± 0.04	0.093	
Gender, male	19 ± 82.6%	11 ± 44.0%	0.006	ASD > TD
Handedness, right-handed	15 ± 65.2%	21 ± 84.0%	0.133	
Clinical				
RBS-R				
RSM	4.04 ± 4.37	N/A		
IS	11.26 ± 9.60	N/A		
Total	15.30 ± 11.27	N/A		
SRS				
Social Awareness	11.04 ± 2.90	7.56 ± 3.20	< 0.001	ASD < TD
Social Cognition	16.78 ± 5.25	9.44 ± 4.12	< 0.001	ASD < TD
Social Communication	26.87 ± 9.78	9.84 ± 5.25	< 0.001	ASD < TD
Social Motivation	12.26 ± 6.68	6.04 ± 3.35	< 0.001	ASD < TD
Restricted Interests and Repetitive Behavior	12.83 ± 5.93	4.84 ± 3.35	< 0.001	ASD < TD
Total	79.78 ± 25.87	37.72 ± 15.20	< 0.001	ASD < TD
DCDQ				
Fine motor/ handwriting	11.65 ± 3.77	15.16 ± 3.35	0.001	ASD > TD
General coordination	22.96 ± 5.41	29.88 ± 4.33	< 0.001	ASD > TD
Control during movement	18.83 ± 4.91	24.64 ± 5.07	< 0.001	ASD > TD
Total	53.43 ± 10.30	69.68 ± 10.77	< 0.001	ASD > TD

### 3.2 Abnormal between-network FC among autistic children and its relationship with motor coordination

**Figure 3:**
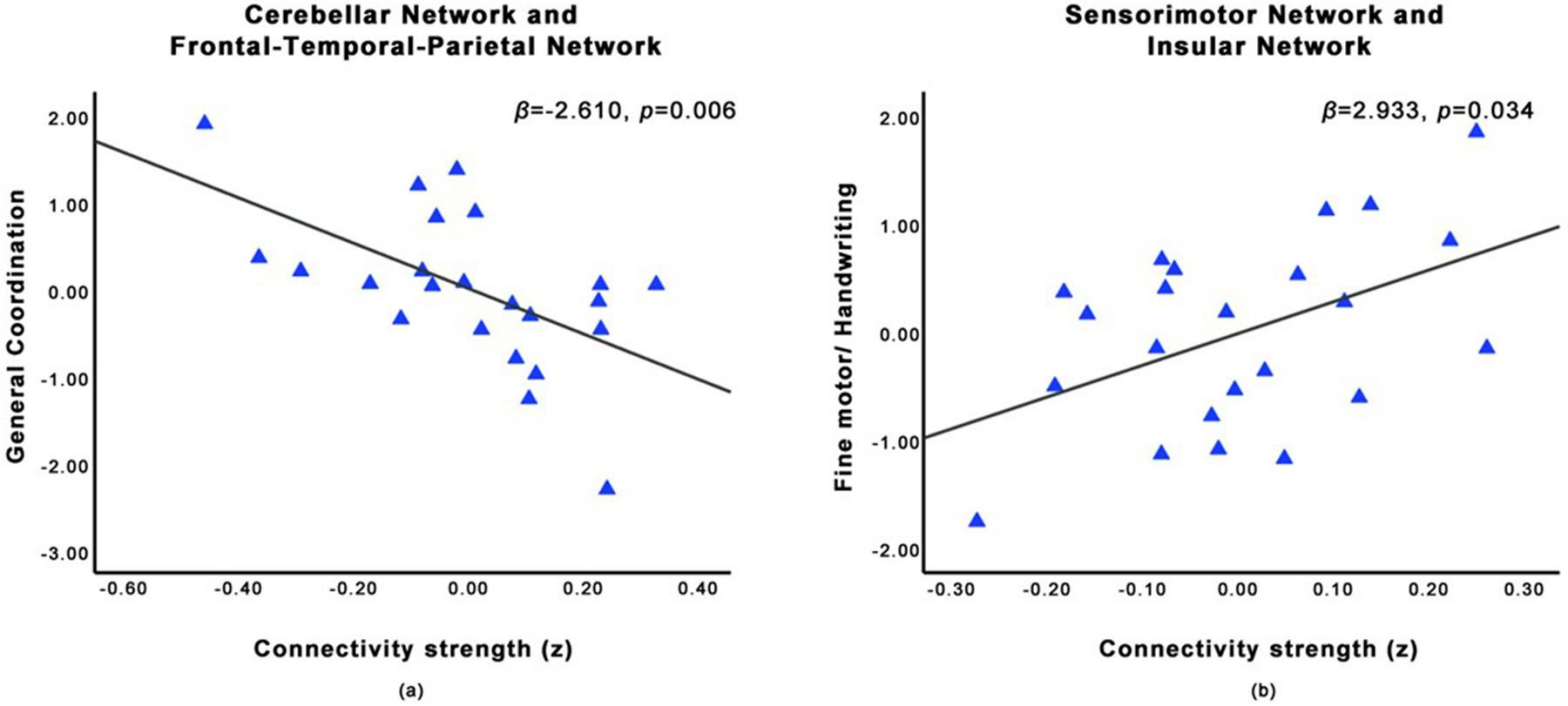
**(a)** Among ASD group, FC between the cerebellar network and frontal-temporal-parietal network was negatively associated with score of general coordination (age and gender as covariates); **(b)** Among ASD group, FC between the sensorimotor network and insular network was positively associated with score of fine motor/handwriting (age, gender and IQ as covariates).

Controlling age and gender, 3 pairs of correlation coefficients revealed significantly decreased between-network FC in children with ASD, relative to TD (*p* < 0.05; Table 2): sensorimotor network and insular network (*t* = −4.612), cerebellar- occipital network and insular network (*t* = −4.180), as well as cerebellar network and frontal-temporal-parietal network (*t* = −3.667). Among the ASD group, the FC between the cerebellar network and frontal-temporal-parietal network was negatively associated with the general coordination score (*β* = −2.610, *p* = 0.006; Figure 3a). Additionally, controlling full-scale IQ, group differences exhibited in 3 pairs of between-network FC (*p* < 0.05; Table 2): sensorimotor network and insular network (*t* = −4.314), cerebellar-occipital network and insular network (*t* = −3.459), as well as sensorimotor network and Lat. Frontal network (*t* = −3.514). The FC between the sensorimotor network and insular network was positively associated with fine motor/handwriting score (*β* = 2.933, *p* = 0.034; Figure 3a).

**Table 2:** Significantly decreased between-network connectivity in children with ASD compared with TD.

Network 1	Network 2	Mean Correlation (z) TD	Mean Correlation (z) ASD	*t*	*p* uncorrected
Age and gender as covariates					
Sensorimotor	Insular	0.384 ± 0.134	0.142 ± 0.153	−4.612	<0.001^a^
Cerebellar-OCC.	Insular	0.212 ± 0.235	−0.093 ± 0.148	−4.180	<0.001^a^
Cerebellar	Frontal-Temporal-Parietal	0.116 ± 0.162	−0.106 ± 0.203	−3.667	<0.001^a^
Age, gender and IQ as covariates					
Sensorimotor	Insular	0.384 ± 0.134	0.142 ± 0.153	−4.314	<0.001^a^
Cerebellar-OCC.	Insular	0.212 ± 0.235	−0.093 ± 0.148	−3.459	<0.01^a^
Sensorimotor	Lat. Frontal	0.073 ± 0.219	−0.131 ± 0.169	−3.514	<0.01^a^

a false discovery rate adjusted *p*-value < 0.05.

### 3.3 Abnormal within-network FC among autistic children and its relationship with motor coordination

**Figure 4:**
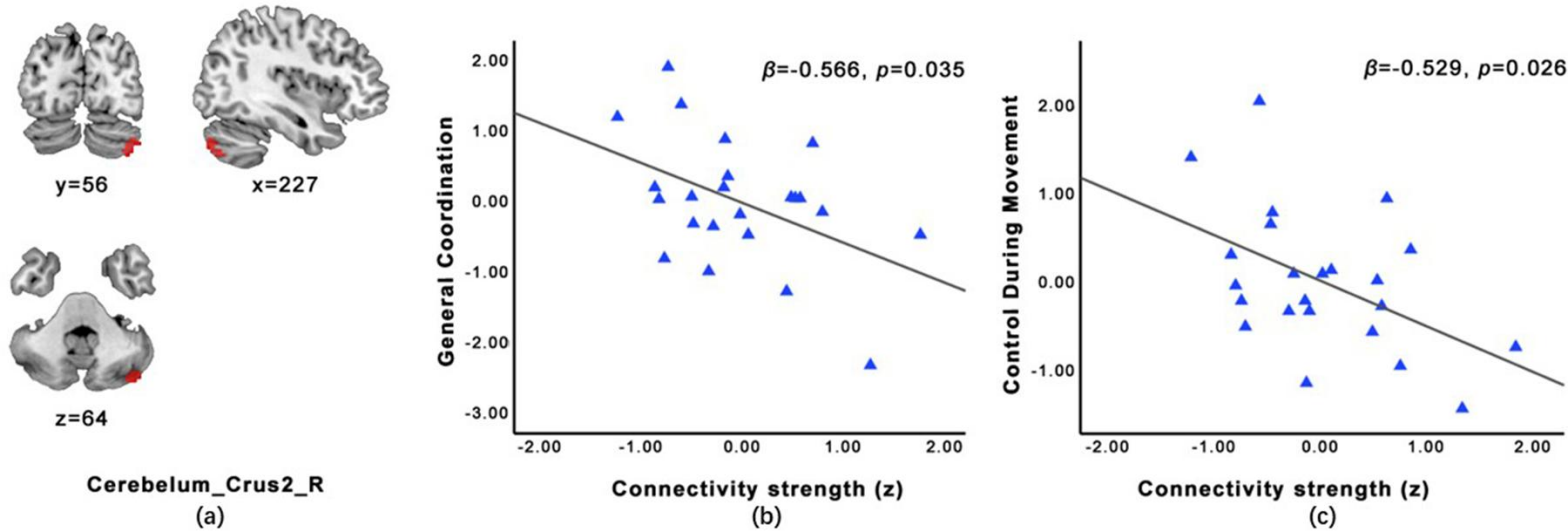
Location of cerebellum crus2 **(a)**. Among ASD children, the intra-FC of the right cerebellar crus II was negatively associated with the score of general coordination (**b**, *β* = -0.566, *p* = 0.035) and control during movement (**c**, *β* = -0.529, *p* = 0.026) age and gender as covariates.

Controlled for age and gender, children with ASD showed significantly increased FC in the right postcentral gyrus of the IC20 (*t* = 4.159, *p* < 0.05), increased FC in the right paracentral lobule of the IC2 (*t* = 4.326, *p* < 0.05), and decreased FC in the right cerebellar crus II of the IC20 (*t* = −4.572, *p* < 0.05) compared with TD group (Table 3). Among ASD children, the intra-FC of the right cerebellar crus II was negatively associated with the score of general coordination (*β* = −0.566, *p* = 0.035) and control during movement (*β* = −0.529, *p* = 0.026; Figure 4). Children with ASD showed significantly increased intra-network FC within the sensory and sensorimotor network (IC20), as evidenced by a cluster of increased connectivity in the right postcentral gyrus (*t* = 4.470, *p* < 0.05; Table 3).

**Table 3:** Significantly increased/decreased FC in neural networks of children with ASD compared with TD.

IC	Network	Increased or decreased (+/-)	Abnormal brain region	MNI (x, y, z)	Voxels	*t*
Age and gender as covariates						
IC20	Sensory	+	Postcentral_R	45, −18, 51	42	4.159
IC2	Sensorimotor	+	Paracentral_Lobule_R	9, −33, 63	92	4.326
IC5	Cerebellar	-	Cerebelum_Crus2_R	36, −78, −45	38	−4.572
Age, gender and IQ as covariates						
IC20	Sensory	+	Postcentral_R	33, −33, 66	59	4.470

False discovery rate adjusted *p*-value < 0.05.

IC: Independent Component. The number denotes the specific component from the group-ICA decomposition. IC20 was identified as the sensory network, IC5 as the cerebellar network, etc.

## 4 Discussion

This study gained an understanding of the relationship between motor coordination impairment and altered within-/between-network FC among autistic children. The work confirmed the important role of the sensory, sensorimotor, and cerebellar networks in the motor coordination of ASD. We also found significant importance of the relationship between the insular network and the sensorimotor network as well as the relationship between the cerebellar network and frontal-temporal-parietal in motor coordination among autistic children.

The cerebellar circuits play an important role by integrating and relaying error information to frontal, parietal, and temporal cortices to modulate motor processing, thus altered cerebellar connectivity may play a key role in sensorimotor defect (11, 42). In our research, the relationship between greater cerebellar-Frontal-Temporal-Parietal FC and worse motor performance among ASD supported this indication. According to Lepping et al., the atypical development of cerebellar–cortical networks may underpin sensorimotor impairments (21), because fronto-parietal-cerebellar networks were involved in the planning of movements, and altered FC may contribute to atypical motor skills (43). This mechanism was also supported by a tb-fMRI study, which found greater FC in ASD in cerebellar–parietal circuits (associated with fundamental sensory and sensorimotor processes) and reduced FC in cerebellar–frontal and cerebellar–temporal circuits (associated with more complex multisensory and cognitive processes) in ASD during visuomotor tasks (11). Wang et al. also found that functional connectivity between the right cerebellum and left inferior frontal gyrus was significantly enhanced after a 12-week basketball training, matching the previous theories (44).

Sedation-induced functional disruption occurs both in specific networks and among the whole brain system (45, 46), especially regions involved in motor and sensory processing (47), which explained significantly decreased between-network FC in children with ASD (sensorimotor and insular network, cerebellar- occipital network and insular network, as well as cerebellar network and frontal-temporal-parietal network), relative to TD. Meanwhile, as chloral hydrate was only used in the ASD group, the mentioned network was reduced compared with the TD group is not surprising.

Increased within-network FC in the sensory and sensorimotor network among ASD was consistent with previous studies (48, 49), including greater FC in the precentral and postcentral gyri (23). It indicated that enhanced within-network FC may result in over-sensitivity to sensorimotor stimuli, and limit interactions among networks (23), thus contributing to the motor dysfunction of ASD. There were controversial findings about cerebellum connectivity in previous studies. Increased (7) and decreased within-cerebellum connectivity (11) in autistic children were both reported. Haghighat et al. found increased within-cerebellum in autistic children and increased/decreased connectivity in autistic adolescents and adults (50). The difference may result from different age-range and methods applied. We also found that greater intra-cerebellar FC was related to worse motor performance, indicating that cerebellar overconnectivity may interfere with motion processing among autistic individuals. Cerebellar crus II is involved in integrating complex and multi-sensory information during goal-directed activities, whose dysfunction may contribute to impaired motor coordination function (42).

Our results confirmed the important role of the insular network in motor coordination among ASD, as we found FC between the insular network and sensorimotor network was decreased, which was correlated with fine motor/handwriting skills of the ASD group. The insula has been consistently identified as a locus of hypoactivity and dysfunctional connectivity in ASD, contributing to multisensory dysfunction (51). This is supported by previous researches (52, 53), which found reduced functional connectivity in ASD between bilateral posterior insular cortices (involved in sensorimotor processing) with ventral and dorsal somatosensory cortices. Previous research focused on its association with impaired interoceptive awareness (53, 54), while recent researchers suggested that interoception interacts with motor actions, which helps reflexive forms of action control (55). We also found decreased FC between the insular network and cerebellar-occipital network. It further supported the claim that autistic children favored proprioceptive over visual feedback during motor learning due to impaired ability to process visual information (18, 56, 57).

To theoretically contextualize the potential impact of sedation, we compared our findings in the sedated ASD group against the non-sedated TD group, while referencing the established literature on sedative effects. Sedative agents like chloral hydrate are generally associated with a global reduction of functional connectivity and brain activity (58). Interestingly, our results revealed a more complex pattern, including increases in intra-network FC within sensory and sensorimotor networks. This divergence from the expected suppressive effect of sedation suggests that the observed alterations may not be a passive pharmacological artifact alone. Instead, this finding suggests that sedative and the intrinsic neurobiology of ASD may have different interactions than in the neurotypical population (59). Future studies explicitly testing this potential variation in interaction are needed to clarify whether or how non-sedated TD data can be leveraged as a comparison (60.

## 5 Limitations

A primary limitation of this study is the use of chloral hydrate sedation for the ASD group, while the TD children were scanned without sedation. This differential treatment is a significant confound, as anesthetic agents are known to alter functional connectivity, particularly within sensory, motor, and higher-order cognitive networks (61). For instance, studies have shown that general anesthetics can produce a robust, state-dependent reduction of neuronal interactions, with a preferential effect on feedback pathways within sensory cortices (62). Therefore, the observed differences in within- and between-network FC between the ASD and TD groups should be interpreted with caution, as they may stem from a confluence of intrinsic neural traits associated with ASD and acute, sedation-induced alterations in neural activity.

However, the brain-behavior analyses conducted specifically within the ASD group is less susceptible to this confound. Since all ASD participants were scanned under the same sedated condition, the significant correlations we found between aberrant FC and poorer motor coordination scores are unlikely to be explained by sedation alone. These correlations suggest that the identified neural circuits are meaningfully involved in motor coordination in ASD, irrespective of the acute pharmacological state. Future studies employing non-sedated scanning protocols or examining baseline sedation effects in TD populations are warranted to fully disentangle these effects and validate our findings.

This study should be interpreted in the context of the following limitations. Firstly, the sample size, while comparable to previous similar neuroimaging studies of ASD, may limit the statistical power and generalizability of the findings. Secondly, the gender imbalance in the ASD group, though reflective of the disorder's epidemiology, necessitates caution in extending these findings to females with ASD. Future studies with larger, sex-balanced or female-only samples are required to confirm and extend our results. Third, motor coordination was assessed using a parent-reported questionnaire (DCDQ) rather than a performance-based measure. While the DCDQ provides valuable information on motor skills in daily life and has demonstrated good validity, it may be subject to parental perception bias. Future studies would benefit from combining parent reports with objective motor assessments such as the Movement Assessment Battery for Children-2nd edition (MABC-2) or kinematic testing. These tools can provide highly precise, quantifiable data on specific motor components (e.g., manual dexterity, aiming and catching, balance) and movement kinematics, which would complement the ecological information provided by parent-reported questionnaires like the DCDQ, and longitudinal studies are needed to determine causal pathways.

## 6 Conclusion

In the present study, we found altered cerebellar, sensorimotor, and insula circuits among autistic children, and further confirmed significant relationships between altered within-/between-network FC and motor coordination impairment among autistic children, including increased intra-cerebellar FC, cerebellar-frontal-temporal-parietal network FC and decreased sensorimotor-insular network FC. These findings partly reveal the neuroimage traits of motor coordination impairment in ASD, in particular, the insular network in interoception and motor processing among ASD has been confirmed, and we also propose that the motor dysfunction of autistic individuals were related to impaired information integrating, relaying, and visual feedback during movement. These findings indicated atypical FC patterns in autistic children, which could provide evidence for the design of motor-related intervention for children with ASD. Future studies should adopt longitudinal designs to track the co-development of functional connectivity and motor skills in ASD from an early age. Furthermore, advanced analytical approaches, such as dynamic causal modeling (DCM), are warranted to investigate the effective (directional) connectivity within the identified cerebellar-insular-sensorimotor pathways, moving beyond correlation to better infer causal influences.

## Data Availability

The raw data supporting the conclusions of this article will be made available by the authors, without undue reservation.

## References

[1] Ketcheson LR Pitchford EA Wentz CF. The relationship between developmental coordination disorder and concurrent deficits in social communication and repetitive behaviors among children with autism Spectrum disorder. Autism Res. (2021) 14(4):804–16. 10.1002/aur.2469 33421296

[2] Leonard HC Hill EL. Executive difficulties in developmental coordination disorder: methodological issues and future directions. Curr Dev Disord Rep. (2015) 2(2):141–9. 10.1007/s40474-015-0044-8

[3] Uljarević M Hedley D Alvares GA Varcin KJ Whitehouse AJO. Relationship between early motor milestones and severity of restricted and repetitive behaviors in children and adolescents with autism spectrum disorder. Autism Res. (2017) 10(6):1163–8. 10.1002/aur.1763 28301081

[4] Taverna EC Huedo-Medina TB Fein DA Eigsti I-M. The interaction of fine motor, gesture, and structural language skills: the case of autism spectrum disorder. Res Autism Spectr Disord. (2021) 86:101824. 10.1016/j.rasd.2021.101824 34306180 PMC8294070

[5] Jasmin E Couture M McKinley P Reid G Fombonne E Gisel E. Sensori-motor and daily living skills of preschool children with autism spectrum disorders. J Autism Dev Disord. (2009) 39(2):231–41. 10.1007/s10803-008-0617-z 18629623

[6] Fournier KA Hass CJ Naik SK Lodha N Cauraugh JH. Motor coordination in autism spectrum disorders: a synthesis and meta-analysis. J Autism Dev Disord. (2010) 40(10):1227–40. 10.1007/s10803-010-0981-3 20195737

[7] Allen G Courchesne E. Differential effects of developmental cerebellar abnormality on cognitive and motor functions in the cerebellum: an fMRI study of autism. Am J Psychiatry. (2003) 160(2):262–73. 10.1176/appi.ajp.160.2.262 12562572

[8] Mostofsky SH Powell SK Simmonds DJ Goldberg MC Caffo B Pekar JJ. Decreased connectivity and cerebellar activity in autism during motor task performance. Brain J Neurol. (2009) 132(Pt 9):2413–25. 10.1093/brain/awp088 PMC2732264 19389870

[9] Hanaie R Mohri I Kagitani-Shimono K Tachibana M Matsuzaki J Hirata I Aberrant cerebellar-cerebral functional connectivity in children and adolescents with autism Spectrum disorder. Front Hum Neurosci. (2018) 12:454. 10.3389/fnhum.2018.00454 30483084 PMC6243023

[10] Thompson A Murphy D Dell’Acqua F Ecker C McAlonan G Howells H Impaired communication between the motor and somatosensory homunculus is associated with poor manual dexterity in autism Spectrum disorder. Biol Psychiatry. (2017) 81(3):211–9. 10.1016/j.biopsych.2016.06.020 27639500 PMC5227100

[11] Wang Z Sweeney JA Gong Q Lui S Mosconi MW. Resting-State brain network dysfunctions associated with visuomotor impairments in autism Spectrum disorder. Front Integr Neurosci. (2019) 13:17. 10.3389/fnint.2019.00017 31213995 PMC6554427

[12] Villalobos ME Mizuno A Dahl BC Kemmotsu N Müller R-A. Reduced functional connectivity between V1 and inferior frontal cortex associated with visuomotor performance in autism. NeuroImage. (2005) 25(3):916–25. 10.1016/j.neuroimage.2004.12.022 15808991 PMC3319340

[13] Unruh KE Martin LE Magnon G Vaillancourt DE Sweeney JA Mosconi MW. Cortical and subcortical alterations associated with precision visuomotor behavior in individuals with autism spectrum disorder. J Neurophysiol. (2019) 122(4):1330–41. 10.1152/jn.00286.2019 31314644 PMC6843107

[14] Travers BG Bigler ED Tromp DPM Adluru N Destiche D Samsin D Brainstem white matter predicts individual differences in manual motor difficulties and symptom severity in autism. J Autism Dev Disord. (2015) 45(9):3030–40. 10.1007/s10803-015-2467-9 26001365 PMC4554823

[15] Travers BG Kana RK Klinger LG Klein CL Klinger MR. Motor learning in individuals with autism spectrum disorder: activation in superior parietal lobule related to learning and repetitive behaviors. Autism Res. (2015) 8(1):38–51. 10.1002/aur.1403 25258047

[16] Müller RA Pierce K Ambrose JB Allen G Courchesne E. Atypical patterns of cerebral motor activation in autism: a functional magnetic resonance study. Biol Psychiatry. (2001) 49(8):665–76. 10.1016/S0006-3223(00)01004-0 11313034

[17] Müller R Kleinhans N Kemmotsu N Pierce K Courchesne E. Abnormal variability and distribution of functional maps in autism: an FMRI study of visuomotor learning. Am J Psychiatry. (2003) 160(10):1847–62. 10.1176/appi.ajp.160.10.1847 14514501

[18] Marko MK Crocetti D Hulst T Donchin O Shadmehr R Mostofsky SH. Behavioural and neural basis of anomalous motor learning in children with autism. Brain J Neurol. (2015) 138(Pt 3):784–97. 10.1093/brain/awu394 PMC4339776 25609685

[19] Duffield TC Trontel HG Bigler ED Froehlich A Prigge MB Travers B Neuropsychological investigation of motor impairments in autism. J Clin Exp Neuropsychol. (2013) 35(8):867–81. 10.1080/13803395.2013.827156 23985036 PMC3907511

[20] Green RR Bigler ED Froehlich A Prigge MBD Zielinski BA Travers BG Beery VMI and brain volumetric relations in autism Spectrum disorder. J Pediatr Neuropsychol. (2019) 5(3):77–84. 10.1007/s40817-019-00069-z 32953403 PMC7497806

[21] Lepping RJ McKinney WS Magnon GC Keedy SK Wang Z Coombes SA Visuomotor brain network activation and functional connectivity among individuals with autism spectrum disorder. Hum Brain Mapp. (2022) 43(2):844–59. 10.1002/hbm.25692 34716740 PMC8720186

[22] Brieber S Herpertz-Dahlmann B Fink GR Kamp-Becker I Remschmidt H Konrad K. Coherent motion processing in autism spectrum disorder (ASD): an fMRI study. Neuropsychologia. (2010) 48(6):1644–51. 10.1016/j.neuropsychologia.2010.02.007 20153764

[23] Uddin LQ Supekar K Lynch CJ Khouzam A Phillips J Feinstein C Salience network-based classification and prediction of symptom severity in children with autism. JAMA Psychiatry. (2013) 70(8):869–79. 10.1001/jamapsychiatry.2013.104 23803651 PMC3951904

[24] Canario E Chen D Biswal B. A review of resting-state fMRI and its use to examine psychiatric disorders. Psychoradiology. (2021) 1(1):42–53. 10.1093/psyrad/kkab003 38665309 PMC10917160

[25] Fox MD Raichle ME. Spontaneous fluctuations in brain activity observed with functional magnetic resonance imaging. Nature reviews. Neuroscience. (2007) 8(9):700–11. 10.1038/nrn2201 17704812

[26] Lee MH Smyser CD Shimony JS. Resting-state fMRI: a review of methods and clinical applications. AJNR Am J Neuroradiol. (2013) 34(10):1866–72. 10.3174/ajnr.A3263 22936095 PMC4035703

[27] Fukunaga M Horovitz SG van Gelderen P de Zwart JA Jansma JM Ikonomidou VN Large-amplitude, spatially correlated fluctuations in BOLD fMRI signals during extended rest and early sleep stages. Magn Reson Imaging. (2006) 24(8):979–92. 10.1016/j.mri.2006.04.018 16997067

[28] Smith SM Fox PT Miller KL Glahn DC Fox PM Mackay CE Correspondence of the brain’s functional architecture during activation and rest. Proc Natl Acad Sci U S A. (2009) 106(31):13040–5. 10.1073/pnas.0905267106 19620724 PMC2722273

[29] Calhoun VD Liu J Adali T. A review of group ICA for fMRI data and ICA for joint inference of imaging, genetic, and ERP data. NeuroImage. (2009) 45(1 Suppl):S163–72. 10.1016/j.neuroimage.2008.10.057 19059344 PMC2651152

[30] Li C Yuan H Urbano D Cha YH Ding L. ICA On sensor or source data: a comparison study in deriving resting state networks from EEG. Annual International Conference of the IEEE Engineering in Medicine and Biology Society. IEEE Engineering in Medicine and Biology Society. Annual International Conference, 2017 (2017). p. 3604–7 10.1109/EMBC.2017.8037637 29060678

[31] Koch W Teipel S Mueller S Buerger K Bokde ALW Hampel H Effects of aging on default mode network activity in resting state fMRI: does the method of analysis matter? NeuroImage. (2010) 51(1):280–7. 10.1016/j.neuroimage.2009.12.008 20004726

[32] Wilson BN Kaplan BJ Crawford SG Campbell A Dewey D. Reliability and validity of a parent questionnaire on childhood motor skills. Am J Occup Ther. (2000) 54(5):484–93. 10.5014/ajot.54.5.484 11006808

[33] Albajara Sáenz A Villemonteix T Van Schuerbeek P Baijot S Septier M Defresne P Motor abnormalities in attention-deficit/hyperactivity disorder and autism Spectrum disorder are associated with regional grey matter volumes. Front Neurol. (2021) 12:666980. 10.3389/fneur.2021.666980 34017307 PMC8129495

[34] Wilson BN Crawford SG Green D Roberts G Aylott A Kaplan BJ. Psychometric properties of the revised developmental coordination disorder questionnaire. Phys Occup Ther Pediatr. (2009) 29(2):182–202. 10.1080/01942630902784761 19401931

[35] Wang J Xia M Liao X Evans A. GRETNA: a graph theoretical network analysis toolbox for imaging connectomics. Front Hum Neurosci. (2015) 9:386. 10.3389/fnhum.2015.00386 26175682 PMC4485071

[36] Himberg J Hyvärinen A Esposito F. Validating the independent components of neuroimaging time series via clustering and visualization. NeuroImage. (2004) 22(3):1214–22. 10.1016/j.neuroimage.2004.03.027 15219593

[37] Muetzel RL Blanken LME Thijssen S van der Lugt A Jaddoe VWV Verhulst FC Resting-state networks in 6-to-10 year old children. Hum Brain Mapp. (2016) 37(12):4286–300. 10.1002/hbm.23309 27417416 PMC6867466

[38] Rinat S Izadi-Najafabadi S Zwicker JG. Children with developmental coordination disorder show altered functional connectivity compared to peers. Neuroimage Clin. (2020) 27:102309. 10.1016/j.nicl.2020.102309 32590334 PMC7320316

[39] Meda SA Gill A Stevens MC Lorenzoni RP Glahn DC Calhoun VD Differences in resting-state functional magnetic resonance imaging functional network connectivity between schizophrenia and psychotic bipolar probands and their unaffected first-degree relatives. Biol Psychiatry. (2012) 71(10):881–9. 10.1016/j.biopsych.2012.01.025 22401986 PMC3968680

[40] Lam KSL Aman MG. The repetitive behavior scale-revised: independent validation in individuals with autism spectrum disorders. J Autism Dev Disord. (2007) 37(5):855–66. 10.1007/s10803-006-0213-z 17048092

[41] Cen C Liang Y-Y Chen Q-R Chen K-Y Deng H-Z Chen B-Y Investigating the validation of the Chinese mandarin version of the social responsiveness scale in a mainland China child population. BMC Psychiatry. (2017) 17(1):51. 10.1186/s12888-016-1185-y 28166747 PMC5292795

[42] D'Mello AM Stoodley CJ. Cerebro-cerebellar circuits in autism spectrum disorder. Front Neurosci. (2015) 9:408. 10.3389/fnins.2015.00408 26594140 PMC4633503

[43] Hadders-Algra M. Emerging signs of autism spectrum disorder in infancy: putative neural substrate. Dev Med Child Neurol. (2022) 64(11):1344–50. 10.1111/dmcn.15333 35801808 PMC9796067

[44] Wang J Cai K-L Liu Z-M Herold F Zou L Zhu L-N Effects of Mini-basketball training program on executive functions and core symptoms among preschool children with autism Spectrum disorders. Brain Sci. (2020) 10(5):263. 10.3390/brainsci10050263 32365853 PMC7287705

[45] Schröter MS Spoormaker VI Schorer A Wohlschläger A Czisch M Kochs EF Spatiotemporal reconfiguration of large-scale brain functional networks during propofol-induced loss of consciousness. J Neurosci. (2012) 32(37):12832–40. 10.1523/JNEUROSCI.6046-11.2012 22973006 PMC6703804

[46] Schrouff J Perlbarg V Boly M Marrelec G Boveroux P Vanhaudenhuyse A Brain functional integration decreases during propofol-induced loss of consciousness. NeuroImage. (2011) 57(1):198–205. 10.1016/j.neuroimage.2011.04.020 21524704

[47] Wei Z Alcauter S Jin K Peng Z-w Gao W. Graph theoretical analysis of sedation’s effect on whole brain functional system in school-aged children. Brain Connect. (2013) 3(2):177–89. 10.1089/brain.2012.0125 23294031 PMC3634152

[48] Ahmadi SMM Mohajeri N Soltanian-Zadeh H. Connectivity Abnormalities in Autism spectrum Disorder Patients: A Resting State fMRI Study. Tehran: IEEE (2014).

[49] Wang J Duan X Chen H He C Zhai J Wu L Atypical resting-state functional connectivity of intra/inter-sensory networks is related to symptom severity in young boys with autism Spectrum disorder. Front Physiol. (2021) 12:626338. 10.3389/fphys.2021.626338 33868000 PMC8044873

[50] Haghighat H Mirzarezaee M Araabi BN Khadem A. Functional networks abnormalities in autism Spectrum disorder: age-related hypo and hyper connectivity. Brain Topogr. (2021) 34(3):306–22. 10.1007/s10548-021-00831-7 33905003

[51] Gogolla N. The insular cortex. Curr Biol. (2017) 27(12):R580–6. 10.1016/j.cub.2017.05.010 28633023

[52] Deen B Pitskel NB Pelphrey KA. Three systems of insular functional connectivity identified with cluster analysis. Cerebral Cortex (New York, N.Y.: 1991). (2011) 21(7):1498–506. 10.1093/cercor/bhq186 21097516 PMC3116731

[53] Ebisch SJH Gallese V Willems RM Mantini D Groen WB Romani GL Altered intrinsic functional connectivity of anterior and posterior insula regions in high-functioning participants with autism spectrum disorder. Hum Brain Mapp. (2011) 32(7):1013–28. 10.1002/hbm.21085 20645311 PMC6870194

[54] Failla MD Peters BR Karbasforoushan H Foss-Feig JH Schauder KB Heflin BH Intrainsular connectivity and somatosensory responsiveness in young children with ASD. Mol Autism. (2017) 8:25. 10.1186/s13229-017-0143-y 28630661 PMC5470196

[55] Marshall AC Gentsch A Schütz-Bosbach S. The interaction between interoceptive and action states within a framework of predictive coding. Front Psychol. (2018) 9:180. 10.3389/fpsyg.2018.00180 29515495 PMC5826270

[56] Haswell CC Izawa J Dowell LR Mostofsky SH Shadmehr R. Representation of internal models of action in the autistic brain. Nat Neurosci. (2009) 12(8):970–2. 10.1038/nn.2356 19578379 PMC2740616

[57] Mosconi MW Mohanty S Greene RK Cook EH Vaillancourt DE Sweeney JA. Feedforward and feedback motor control abnormalities implicate cerebellar dysfunctions in autism spectrum disorder. J Neurosci. (2015) 35(5):2015–25. 10.1523/JNEUROSCI.2731-14.2015 25653359 PMC4315832

[58] Yang W Jian M Wang X Zhou Y Liang Y Chen Y Dynamic cortical connectivity during propofol sedation in glioma patients. J Neurosurg Anesthesiol. (2025) 37(2):166–73. 10.1097/ANA.0000000000000964 38577956

[59] Nazari R Salehi M Shoeibi A. An explainable connectome convolutional transformer for multimodal autism Spectrum disorder classification. Int J Neural Syst. (2025) 35(8):2550043. 10.1142/S0129065725500431 40621646

[60] Bedford SA Lai M-C Lombardo MV Chakrabarti B Ruigrok A Suckling J Brain-Charting autism and attention-deficit/hyperactivity disorder reveals distinct and overlapping neurobiology. Biol Psychiatry. (2025) 97(5):517–30. 10.1016/j.biopsych.2024.07.024 39128574

[61] Li X Wang L Qin B Zhang Y Zhou Z Qin Y A sleeping rs-fMRI study of preschool children with autism Spectrum disorders. Curr Med Imaging. (2020) 16(7):921–7. 10.2174/1573405616666200510003144 32386497

[62] Hudetz AG Pillay S Wang S Lee H. Desflurane anesthesia alters cortical layer-specific hierarchical interactions in rat cerebral Cortex. Anesthesiology. (2020) 132(5):1080–90. 10.1097/ALN.0000000000003179 32101967 PMC8190972

