# Supplementary material for: The association between motor coordination impairment and altered functional connectivity among autistic children
Source: Front Pediatr. 2026 Feb 10;14:1711271. doi: 10.3389/fped.2026.1711271 (PMC12930269; doi:10.3389/fped.2026.1711271)
Supplement: Supplementary file 1 [file Datasheet1.pdf]

eTable 1 Literature review of studies on the relationship between motor dysfunction and neuroimaging changes

Author (year)	Title	Study design	sample size	Age of sample	Neuroimaging result	Neuroimaging method	Motor function	Motor function measurement tool	ASD diagnosis tool	Main findings	Limitations
**Lepping et al. 2021**	Visuomotor brain network activation and functional connectivity among individuals with autism spectrum disorder	Cross-sectional	ASD=27, TD=30	9-35 years	BOLD activation FC	tb-fMRI	precision motor behaviors	behavioral force measures	ADI-R ADOS-2 DSM-IV-TR	Visuomotor deficits in ASD are associated with atypical activation and functional connectivity of posterior parietal, premotor, and striatal circuits	younger and more severely impaired individuals with comorbidity; IQ and gender were not matched
**Wang et al. 2019**	Resting-State Brain Network Dysfunctions Associated With Visuomotor Impairments in Autism Spectrum Disorder	Cross-sectional	ASD=23, TD=16	10–33 years	ALFF	rs-fMRI tb-fMRI	precision force control	visually guided precision gripping	ADI-R ADOS DSM-V	Greater ALFF in cerebellar vermis VI was associated with less visuomotor variability in ASD.	Small sample size of individuals who completed both tb- and rs-fMRI runs.

Author (year)	Title	Study design	sample size	Age of sample	Neuroimaging result	Neuroimaging method	Motor function	Motor function measurement tool	ASD diagnosis tool	Main findings	Limitations
**Unruh et al. 2019**	Cortical and subcortical alterations associated with precision visuomotor behavior in individuals with autism spectrum disorder	Cross-sectional	ASD=20, TD=18	14–33 years	BOLD activation	tb-fMRI	force variability	grip force fMRI task	ADI-R ADOS DSM-V	The BOLD signal for multiple cortical and subcortical regions was associated with force variability, including motor and premotor cortex, posterior parietal cortex, extrastriate cortex, putamen, and cerebellum.	Relatively small sample size; not allow for comparison of potential sex differences; Analyses rely on qualitative ratings of behavior.

Author (year)	Title	Study design	sample size	Age of sample	Neuroimaging result	Neuroimaging method	Motor function	Motor function measurement tool	ASD diagnosis tool	Main findings	Limitations
**Travers et al. 2015**	Motor Learning in Individuals With Autism Spectrum Disorder: Activation in Superior Parietal Lobule Related to Learning and Repetitive Behaviors	Cross-sectional	ASD=15, TD=15, all male	ASD: 20.81±3.98 years TD: 21.41±2.85 years	BOLD activation	tb-fMRI	motor learning	serial reaction time (SRT) task	ADI-R	decreased activation in the right superior parietal lobule (SPL) and right precuneus during learning among ASD	limited spatial resolution.
**Brieber et al. 2010**	Coherent motion processing in autism spectrum disorder (ASD): an fMRI study	Cross-sectional	ASD=15, TD=15, all male	13–19 years	BOLD activation	tb-fMRI	abnormal motion processing	coherent motion detection task	ICD-10 DSM-IV ADOS-G ADI-R	ASD showed increased brain activation in the left primary visual cortex. A significant interaction effect between group and condition in the right	1. Limited sample size.<br>2. No significant behavioral differences between the control and the ASD group.

Author (year)	Title	Study design	sample size	Age of sample	Neuroimaging result	Neuroimaging method	Motor function	Motor function measurement tool	ASD diagnosis tool	Main findings	Limitations
										superior parietal cortex only in the control group.	
**Mostofsky et al. 2009**	Decreased connectivity and cerebellar activity in autism during motor task performance	Cross-sectional	ASD=13, TD=13	8–12 years	BOLD activation FC	tb-fMRI	simple motor execution	finger-sequencing paradigm	DSM-IV ADI-R ADOS-G	The TD group showed greater activation in the ipsilateral anterior cerebellum, while the ASD group showed greater activation in the SMA.	the potential for the behavioural differences between groups to have driven the observed neural activation.
**Villalobos et al. 2005**	Reduced functional connectivity between V1 and inferior frontal cortex associated	Cross-sectional	ASD=8, TD=8, all male	15–43 years	FC	tb-fMRI	visuomotor performance	a four-button press device	CARS ADI-R DSM	Functional connectivity between primary visual area 17 and	Small sample size.

Author (year)	Title	Study design	sample size	Age of sample	Neuroimaging result	Neuroimaging method	Motor function	Motor function measurement tool	ASD diagnosis tool	Main findings	Limitations
	with visuomotor performance in autism									inferior frontal cortex was significantly reduced in the autism group.	
**Allen et al. 2003**	Differential effects of developmental cerebellar abnormality on cognitive and motor functions in the cerebellum: an FMRI study of autism	Cross-sectional	ASD=8, TD=8	13–39 years	activation volume & activation extent	tb-fMRI	cognitive and motor functions	motor, sensory and attention tasks	DSM-IV ADI-R	autistic individuals showed significantly greater cerebellar motor activation and significantly less cerebellar attention activation.	Limitations in statistical power and the presence of outliers.
**Müller et al.2003**	Abnormal variability and distribution of functional maps in autism: An fMRI study of	Cross-sectional	ASD=8, TD=8, all male	15–41 years	BOLD activation	tb-fMRI	visually driven motor sequence learning	visually paced finger movement	DSM-IVCARSADI-R	Superior parietal activations were less pronounced but prefrontal	Limited sample size.

Author (year)	Title	Study design	sample size	Age of sample	Neuroimaging result	Neuroimaging method	Motor function	Motor function measurement tool	ASD diagnosis tool	Main findings	Limitations
	visuomotor learning									cortex and more posterior parietal loci showed greater activation in ASD.	
**Müller et al. 2001**	Atypical patterns of cerebral motor activation in autism: a functional magnetic resonance study	Cross-sectional	ASD=8, TD=8, all male	15–41 years	BOLD activation	tb-fMRI	visually paced finger movement	visually paced finger movement task	DSM-IV CARS ADI-R	Greater activation (or reduced deactivation) in posterior and prefrontal cortices in the autism group.	Limited sample size.
**Ryuzo Hanaie et al. (2018)**	Aberrant Cerebellar–Cerebral Functional Connectivity in Children and Adolescents With Autism	Cross-sectional	ASD=16, TD=20	6-15 years	FC	rs-fMRI	motor coordination	Movement Assessment Battery for Children-2 Checklist	ADOS-G DSM-IV	FC value between the vermis VI and right MdFG was negatively correlated	Heterogeneity of the participants with ASD in the replication dataset; relatively small sample size.

Author (year)	Title	Study design	sample size	Age of sample	Neuroimaging result	Neuroimaging method	Motor function	Motor function measurement tool	ASD diagnosis tool	Main findings	Limitations
	Spectrum Disorder									with total test score on the MABC-2.	
**Thompson et al. 2017**	Impaired Communication Between the Motor and Somatosensory Homunculus Is Associated With Poor Manual Dexterity in Autism Spectrum Disorder	Cross-sectional	ASD=60, TD=60	18-45 years	FA MD	DTI	manual dexterity	The Purdue Pegboard Test	ICD-10	Disruption of the S1-M1 connections was associated with precision grasping impairments in individuals with ASD.	Sample is restricted to a high-functioning ASD group.
**Travers et al. 2015**	Brainstem white matter predicts individual differences in manual motor difficulties and symptom severity in autism	Cross-sectional	ASD=67, TD=42, all male	5-33 years	FA MD	DTI	grip strength finger tapping speed	hand dynamometer manual finger tapping board	ADI-R ADOS-G DSM-IV	Weaker grip strength predicted more severe autism symptoms. FAof the brainstem's	Indices of motor function were limited to grip strength and finger tapping.

Author (year)	Title	Study design	sample size	Age of sample	Neuroimaging result	Neuroimaging method	Motor function	Motor function measurement tool	ASD diagnosis tool	Main findings	Limitations
										corticospinal tract predicted grip strength and autism symptom severity	
**Green et al. 2019**	Beery VMI and Brain Volumetric Relations in Autism Spectrum Disorder	Cross-sectional	ASD=41, TD=27, all male	3–23 years	brain volume	sMRI	Visual-Motor Integration	Beery-Buktenica Developmental Test of Visual-Motor Integration (Beery VMI)	ADI-R ADOS-G	Significant correlations were observed in TD between VMI and total precentral gyrus/ frontal lobe gray matter volume.	The sample did not include females; not investigate white matter integrity measures to understand the functional connectivity
**Marko et al. 2015**	Behavioural and neural basis of anomalous motor learning in children with autism	Cross-sectional	ASD=20, TD=20	8–12 years	cerebellum volume	sMRI	learning control of reaching	‘shoot through a target’ game	ADOS ADI-R	The anterior cerebellum were smaller than normal in	The atlas was developed from anatomical data of healthy adults.

Author (year)	Title	Study design	sample size	Age of sample	Neuroimaging result	Neuroimaging method	Motor function	Motor function measurement tool	ASD diagnosis tool	Main findings	Limitations
										ASD, and predicted by the pattern of learning.	
**Duffield et al. 2013**	Neuropsychological investigation of motor impairments in autism	Cross-sectional	ASD=59, TD=33, all male	5–33 years	motor cortex volume	sMRI	motor impairments	strength of grip (SOG), finger tapping test (FTT), and grooved peg-board test (GPT)	ADI-R ADOS DSM-IV	Performance by ASD participants on the GPT and FTT differed significantly from controls. FTT was negatively related to precentral gyrus volume in ASD.	1. The autism sample was all male and comprised of high functioning individuals; not directly assess connectivity; not assess presence of clinical motor signs and their relationship to objective neuropsychological measures of motor functioning.

ASD, autism spectrum disorder; TD, typical development; ADOS, Autism Diagnostic Observation Schedule; ADI-R, Autism Diagnostic Interview-Revised; DSM, Diagnostic and Statistical Manual of Mental Disorders; ICD, International Classification of Diseases; sMRI, structual magnetic resonance imaging; tb-fMRI, task-based functional magnetic resonance imaging; rs-fMRI, resting-state functional magnetic resonance imaging; BOLD, Blood Oxygen Level-Dependent; FC, functional connectivity; ALFF, amplitude of low frequency fluctuations
